# Incidence and risk factors for foot fractures in China: A retrospective population-based survey

**DOI:** 10.1371/journal.pone.0209740

**Published:** 2018-12-26

**Authors:** Song Liu, Yanbin Zhu, Lin Wang, Wei Chen, Xiaolin Zhang, Yingze Zhang

**Affiliations:** 1 Department of Orthopaedic Surgery, the Third Hospital of Hebei Medical University, Shijiazhuang, Hebei, P.R. China; 2 Key laboratory of biomechanics of Hebei Province, Shijiazhuang, Hebei, P.R. China; 3 Department of statistics and epidemiology, Hebei Medical University, Shijiazhuang, Hebei, P.R. China; 4 Chinese Academy of Engineering, Beijing, P.R. China; Cardiff University, UNITED KINGDOM

## Abstract

**Purpose:**

The literature lacks population-based epidemiologic studies on the incidence and risk factors for traumatic foot fractures. The purpose of this study was to update information concerning the incidence of foot fractures in China and to identify associated risk factors.

**Methods:**

All the data on foot fractures were available from the China National Fracture Survey (CNFS), which was conducted between January and May in 2015. A total of 8 provinces, 24 urban cities and 24 rural counties in China were selected, using stratified random sampling and the probability proportional to size method. Individuals who had lived in their current residence for 6 months or longer were personally interviewed about any foot fracture that had occurred in 2014. Questionnaires were completed by every participant for data collection and quality control was accomplished by our research team members. The information included age, gender, height, weight, ethnic group, education, occupation, smoking, alcohol consumption, sleeping time per day, dietary habits and others. Fracture was initially identified by patients' self report and further confirmed by their providing medical records.

**Results:**

A total of 512187 individuals participated in the CNFS. There were 201 patients with foot fractures in 2014. Mean age at the time of fracture was 45.4 years. The incidence rate of foot fractures was 39.2 (95%CI: 33.8–44.7)/100000/year. Fall and traffic accident were the most common causes for foot fractures and over 60% of these occurred at home or on the road. Alcohol consumption, history of previous fracture and average sleep time <7h/d were identified as independent risk factors for foot fractures both in males and females. Cigarette smoking was identified as a significant risk factor for foot fracture in males. For females, BMI >24 kg/m^2^ was a risk factor whilst living in the west region was associated with a lower incidence rate of foot fracture.

**Conclusions:**

The present study shows an incidence of 39.2/100000/year of foot fractures in China. Specific public health policies focusing on decreasing alcohol consumption and encouraging individuals to obtain sufficient sleep should be implemented. Females with a higher BMI should focus more on foot health care, especially in those with history of previous fracture.

## Introduction

Foot fracture is a very commonly seen injury in departments of emergency or orthopaedics [1, 2]. Currently, the literature lacks population-based epidemiologic studies on the incidence of foot fractures based on a complete population. Most reports have concentrated solely on participants from one or several hospitals and regions, or some subgroups and others focused on fracture-type of foot and ankle fractures [1, 3, 4, 5, 6]. Court-Brown et al [7] conducted a retrospective epidemiologic study of adult fractures in Scotland between 2000 and 2001, and found that the incidence of foot fractures was 136.9/100000/year. Kannus et al [6] investigated the epidemiology of fall-related foot fractures in patients aged 50 years and older between 1970 and 2013, and found that the incidence increased from 5.6/100000 in 1970 to 15.0/100000 in 2013. In both studies [6, 7], fractures were counted and confirmed by orthopaedic surgeons and in the latter only patients admitted to hospitals were involved, which undoubtedly under-estimated the incidence of this injury because a certain number foot fracture patients do not require admission for treatment.

Information about epidemiology of foot fractures is of academic interest and is useful in health service research for policy makers. Increased knowledge of factors associated with risk of fracture would ultimately enable us to initiate preventive measures. However, most epidemiologic studies on foot fractures lack information about associated social-economic activities and living habits, and only focus on certain subtypes, such as osteoporosis-related fracture [3, 4, 6]. Furthermore, we have found no studies using population-based design to investigate the incidence and risk factors for foot fractures in China.

The primary objective of the present study was to provide information concerning the incidence of foot fractures in 2014 in China and the second objective was to identify the associated risk factors stratified by gender.

## Methods

The CNFS has been described previously [8], which was registered with the Chinese Clinical Trial Registry, number ChiCTR-EPR-15005878. The CNFS was approved by the Institutional Review Board and ethics committee of the 3^rd^ Hospital of Hebei Medical University. Written informed consent was obtained from each participant before data collection. All the data on foot fractures were available from the CNFS from 8 provinces, 24 urban cities and 24 rural counties of China. The detailed method to sample the participants was described in the reference [8]. Briefly, stratified multistage cluster randomized sampling method was used to recruit subjects in this survey. Within each targeted province (municipalities), sampling was done separately in urban and rural areas. Households were calculated and selected. Only members of eligible families living in their current residence for 6 months or longer were invited for face-to-face interview with our trained research team members. Foot fracture was defined a fracture that involved one or combined sites as follows: the talus, calcaneus, navicular, the cuboid, cuneiforms (medial, middle, and lateral), metatarsals and phalanges, caused by high-energy injury (traffic accidents, fall from height and et al) or low-energy injury (fall from standing height).

A standardized questionnaire was administered by trained research teams for data collection. The detailed information included age, sex, height, weight, Chinese ethnic nationality, residence, education, occupation, lifestyles (sleeping time per day, smoking, alcohol drinking and daily consumption of meat, been product, dairy products) for all participants. Individuals who had foot fractures between Jan 1, and Dec 31, 2014, then must answer a more detailed accessory questionnaire regarding the fracture occurrence date and place, fracture site and injury mechanism. In addition, they were asked to provide medical records of their fractures, including radiographs, diagnostic reports, and medical reports. And if these data were not available, the survey team paid for individual participants to obtain a new radiograph of their reported foot at a local hospital.

Ethnic origins were divided into Han ethnicity and others including all the national minority ethnicity. Region was divided into 3 groups based on geographic position and economic level: eastern, middle and west. Urbanization was divided into 2 groups: 1, rural area (village) and 2, urban areas (other than village).The Body mass index (BMI) was calculated by dividing weight by height squared, and was grouped based the reference criteria suited to Chinese people [8,9]: normal, 18.5–23.9; underweight, <18.5; overweight, 24–27.9; obesity, ≥28. Daily diet and drinking including meat and products, bean products, milk and dairy products were divided into 5 groups based on frequency of consumption: never, always (at least 1 per day), often (1/day-1/week), occasionally (1/week-1/month) and seldom (<1/month). Calcium or Vitamin D supplement was defined as positive if participants acknowledged they received Calcium or Vitamin D-related medicine or nourishment via oral or other approaches at least 1 month before the fracture occurrence. Previous history of fracture was defined as a history of fracture at the any site caused by high- or low-energy injury, including humerus, radius, ulna, femur, tibia, fibula, spine, pelvic ring and acetabulum, hand, foot, scapula, clavicle, and patella.

### Statistical analysis

Incidence rates for foot fractures were estimated for the overall population and for subgroups such as age, occupation, education and et al, stratified by gender. For unordered categorical variables such as occupation, regions, residency category, and ethnic origin, the Chi-square test was used to test the differences in incidence of foot fracture. For ordered categorical variables such as age and education level, we entered the related data as a continuous variable into a univariate logistic regression model to test the difference.

Then, univariate Chi-square test was used to investigate the potential correlations between foot fractures and various factors of interest. Adult patients with foot fractures in 2014 were defined as case group, and adult individuals without fractures of any site in 2014 were defined as control group.

Finally, all potential factors associated with foot fractures were tested for significance in multivariable analysis model, with stepwise logistic regression (backward selection) used, to identify the independent factors for males and females separately. We chose a p-value of <0.05 as the level of significance. Odd ratio (OR) values and corresponding 95% confidence interval (95%CI) were estimated to indicate the correlation intension of risk factor. The Hosmer–Lemeshow test was used to examine goodness-of-fit of this model, and a P value > 0.05 indicated an acceptable fitness. SPSS 19.0 was used to perform all analyses (SPSS Inc, Chicago, Illinois, USA).

## Results

Between January and May in 2015, a total of 512187 individuals were invited to participate in the CNFS. There were 1763 patients with 1833 fractures in 2014. Of them, 201 patients sustained foot fractures, indicating that the incidence rate of traumatic foot fractures in China was 39.2 (95%CI: 33.8–44.7)/100000/year (Table 1).

**Table 1:** National incidence of foot fractures among Chinese population by demographic, socio-economic and geographic factors in 2014.

Items	Sample size	Male		Female		Total	
		Case	Incidence (1/100000)	Case	Incidence (1/100000)	Case	Incidence (1/100000)
**Age (years)**							
0–14	81166	9	20.3(7.1–33.6)	2	5.4	11	13.6(5.5–21.6)
15–44	236206	57	48.2(35.7–60.8)	23	19.5(11.5–27.4)	80	33.9(26.4–41.3)
45–64	138533	54	78.1(57.3–98.9)	35	50.4(33.7–67.1)	89	64.2(50.9–77.6)
65+	56282	12	42.7(18.5–66.8)	9	32(11.1–52.9)	21	37.3(21.4–53.3)
P-value for trend test	512187		<0.001		<0.001		<0.001
**Ethnicity**							
Han nationality	477508	123	50.8(41.8–59.8)	67	28.5(21.6–35.3)	190	39.8(34.1–45.4)
Other nationalities	34679	9	51.1(17.7–84.5)	2	11.7	11	31.7(13–50.5)
P-value for difference test	512187		0.987		0.202		0.464
**Region**							
East	232998	72	60.3(46.4–74.2)	43	37.9(26.5–49.2)	115	49.4(40.3–58.4)
Central	99109	18	36.1(19.4–52.8)	12	24.3(10.6–38.1)	30	30.3(19.4–41.1)
West	180080	42	46.4(32.4–60.5)	14	15.6(7.4–23.8)	56	31.1(23–39.2)
P-value for trend test	512187		0.102		0.010		0.004
**Urbanization**							
Urban area	203101	45	43.8(31–56.6)	33	32.8(21.6–44.1)	78	38.4(29.9–46.9)
Rural area	309086	87	55.4(43.8–67.1)	36	23.7(15.9–31.4)	123	39.8(32.8–46.8)
P-value for trend test	512187		0.201		0.172		0.806
**Occupation**							
Office worker	19467	5	53.5(6.6–100.3)	1	9.9	6	30.8(6.2–55.5)
Farmer	106484	30	61.6(39.6–83.6)	26	45(27.7–62.3)	56	52.6(38.8–66.4)
Manual worker	148650	59	71.3(53.1–89.5)	11	16.7(6.8–26.6)	70	47.1(36.1–58.1)
Retired	30366	8	53.9(16.6–91.2)	4	25.8(0.5–51)	12	39.5(17.2–61.9)
Unemployed	32770	4	41.4(0.8–81.9)	7	30.3(7.9–52.7)	11	33.6(13.7–53.4)
Preschool children	35581	2	10.3	1	6.2	3	8.4
Students	80443	7	16.5(4.3–28.8)	7	18.4(4.8–32)	14	17.4(8.3–26.5)
Other	15974	7	77.3(20–134.5)	1	14.5	8	50.1 (15.4–84.8)
Administrator	42452	10	42.7(16.2–69.1)	11	57.8(23.7–92)	21	49.5(28.3–70.6)
P-value for trend test	512187		0.001		0.007		0.001
**Education**							
Illiterate	74937	21	60.9(34.9–87)	21	51.9(29.7–74.1)	42	56(39.1–73)
Primary school	158970	69	86.0(65.7–106.2)	22	28(16.3–39.6)	91	57.2(45.5–69)
Junior high school	121415	28	45.5(28.7–62.4)	18	30(16.2–43.9)	46	37.9(26.9–48.8)
Senior high school or above	40841	12	55.6(24.1–87)	7	36.4(9.4–63.3)	19	46.5(25.6–67.4)
P-value for trend test	396163		0.026		0.178		0.117

The study included 132 males and 69 female patients with foot fractures and their mean age was 45.4 years (SD, 16.9 years). The incidence was 50.8 (95%CI: 42.2–59.5)/100000/year in males and 27.3 (95%CI: 20.9–33.8)/100000/year in females. Slip, trip or fall was the most common cause for foot factures, and leaded to 40.3% (81/201) of injuries, followed by crushing injuries (55, 27.4%), fall from height (38, 18.9%), traffic accidents (24, 11.9%), and other (3, 1.5%) (Table 2). Most of the fractures occurred at home or on the road around, accounting for 63.2% (127/201) of all the injuries (Table 3).

**Table 2:** The causal mechanisms for foot fractures in China in 2014 (n, %).

Injury Mechanism	Children	Adult (≥15 years)		Total
	(0–14 years)	Male	Female	
Traffic Accident	1(9.1)	15(12.2)	8(11.9)	24(11.9)
Slip, Trip or Fall	6(54.5)	31(25.2)	44(65.7)	81(40.3)
Fall from Heights	2(18.2)	29(23.6)	7(10.4)	38(18.9)
Crushing Injury	2(18.2)	45(36.6)	8(11.9)	55(27.4)
Other	0	3(2.4)	0	3(1.5)
Sum	11(100)	123(100)	67(100)	201(100.0)

**Table 3:** The place of foot fracture occurrence in 2014 (n, %).

Place of fracture occurrence	Children	Adult (≥15 year)		Total
		Male	Female	
Home	5(45.5)	38(30.9)	37(55.2)	80(39.8)
Work unit	1(9.1)	24(19.5)	2(3.0)	27(13.4)
Building site	0	27(22.0)	2(3.0)	29(14.4)
Road	3(27.3)	26(21.1)	18(26.9)	47(23.4)
Expressway	0	2(1.6)	5(7.5)	7(3.5)
School	2(18.2)	2(1.6)	1(1.5)	5(2.5)
Others	0	4(3.3)	2(3.0)	6(3.0)
Sum	11(5.5)	123(61.2)	67(33.3)	201(100.0)

Table 1 presented the population-based incidence rates of foot fractures by individual characteristics and regions for the overall populations. There was no significant difference in incidence between those of Han ethnicity and all other ethnicities combined, nor was there any significant difference according to urbanization either for overall population or any gender (Table 1). Stratified by age, individuals of 45–64 years had highest incidence rate of foot fractures (78.1, 50.4 and 64.2 per 100,000 person-year) in males, females and overall population. The difference of incidence rate by age in males, females and overall population all approach to significance (P<0.001). Stratified by region, east region had the highest incidence rates both in males and females, which was 60.3 and 37.9/100000/year, respectively. Stratified by occupation, the difference of incidence rate in males, females and overall population all approach to significance (P = 0.001; P = 0.007; P = 0.001).

Table 4 summarized the detailed results of univariate Chi-square test for adults (≥15 years). For males, education level, meat and products, cigarette smoking, alcohol consumption, average sleep time <7h/d and previous history of fracture were identified to have significant effect on the occurrence of foot fractures. For females, age, region, BMI, dairy and product, bean product, alcohol consumption, average sleep time <7h/d and previous history of fracture were identified to have significant effect on the occurrence of foot fractures.

**Table 4:** Detailed results of univariate analysis for variables of interest.

Variables	Males (n = 214596)		*P*	Females (n = 214964)		*P*
	Case (%)	Control (%)		Case (%)	Control (%)	
**Age (year)**			0.411			0.017
15–44	57(46.3)	117763(54.9)		23(34.3)	117894(54.9)	
45–64	54(43.9)	68753(32.1)		35(52.2)	69026(32.1)	
> = 65	12(9.8)	27985(13)		9(13.4)	27954(13)	
**Region**			0.276			0.005
Eastern	66(53.7)	97708(45.6)		41(61.2)	95515(44.5)	
Middle	15(12.2)	42176(19.7)		12(17.9)	43454(20.2)	
Western	42(34.1)	74617(34.8)		14(20.9)	75905(35.3)	
**Urbanization**			0.376			0.287
Rural area	44(35.8)	85121(39.7)		31(46.3)	85691(39.9)	
Urban area	79(64.2)	129380(60.3)		36(53.7)	129183(60.1)	
**Ethnicity**			0.951			0.244
Han	115(93.5)	200253(93.4)		65(97)	200621(93.4)	
Other	8(6.5)	14248(6.6)		2(3)	14253(6.6)	
**BMI**			0.055			<0.001
18.5–23.9	70(56.9)	138093(64.4)		30(44.8)	144340(67.2)	
24–27.9	41(33.3)	58184(27.1)		26(38.8)	44780(20.8)	
> = 28	7(5.7)	8363(3.9)		9(13.4)	9367(4.4)	
<18.5	5(4.1)	9861(4.6)		2(3.0)	16387(7.6)	
**Education**			0.017			0.063
Illiterate	21(17.1)	34381(16)		21(31.3)	40393(18.8)	
Primary school	64(52.0)	82327(38.4)		21(31.3)	80597(37.5)	
Junior high school	26(21.1)	68337(31.9)		18(26.9)	66554(31.0)	
Senior high school or above	12(9.8)	29456(13.7)		7(10.4)	27330(12.7)	
**Occupation**			0.710			0.378
Unemployed	4(3.3)	9597(4.5)		7(10.4)	22993(10.7)	
Administrator	10(8.1)	23344(10.9)		11(16.4)	18976(8.8)	
Office worker	5(4.1)	9327(4.3)		1(1.5)	10100(4.7)	
Manual worker	59(48)	82403(38.4)		11(16.4)	65762(30.6)	
Farmer	30(24.4)	48460(22.6)		26(38.8)	57500(26.8)	
Retired	8(6.5)	14777(6.9)		4(6)	15420(7.2)	
Students	0	17580(8.2)		6(9)	17253(8)	
Other	7(5.7)	9013(4.2)		1(1.5)	6870(3.2)	
**Meat and product**			0.026			0.354
Never	0(0)	29(0)		2(3)	2523(1.2)	
Always	56(45.5)	111523(52)		29(43.3)	104977(48.9)	
Often	36(29.3)	65004(30.3)		21(31.3)	65151(30.3)	
Occasionally	22(17.9)	29111(13.6)		8(11.9)	31609(14.7)	
Seldom	9(7.3)	8834(4.1)		7(10.4)	10614(4.9)	
**Dairy and product**			0.260			0.009
Never	59(48.0)	92035(42.9)		31(46.3)	77457(36)	
Always	17(13.8)	31533(14.7)		18(26.9)	38374(17.9)	
Often	19(15.4)	34564(16.1)		8(11.9)	41654(19.4)	
Occasionally	18(14.6)	35378(16.5)		6(9)	37593(17.5)	
Seldom	10(8.1)	20991(9.8)		4(6)	19796(9.2)	
**Bean product**			0.712			0.021
Never	1(0.8)	1388(0.6)		0	1256(0.6)	
Always	27(22.0)	40130(18.7)		23(34.3)	40552(18.9)	
Often	51(41.5)	99663(46.5)		27(40.3)	100770(46.9)	
Occasionally	32(26.0)	50185(23.4)		12(17.9)	50150(23.3)	
Seldom	12(9.8)	23135(10.8)		5(7.5)	22146(10.3)	
**Cigarette smoking**			<0.001			0.421
No	44(35.8)	116858(54.5)		66(98.5)	207794(96.7)	
Yes	79(64.2)	97643(45.5)		1(1.5)	7080(3.3)	
**Alcohol consumption**			<0.001			0.005
No	24(19.5)	100778(47.0)		51(76.1)	188566(87.8)	
Yes	99(80.5)	113723(53.0)		16(23.9)	26308(12.2)	
**Living alone**			0.403			0.116
No	122(99.2)	213745(99.6)		66(98.5)	214208(99.7)	
Yes	1(0.8)	756(0.4)		1(1.5)	666(0.3)	
**Living circumstance**			0.105			0.923
Single-storey house	59(48.0)	85619(39.9)		27(40.3)	84696(39.4)	
House ≤7 storey	56(45.5)	113177(52.8)		34(50.7)	114358(53.2)	
House >7 storey	8(6.5)	15705(7.3)		6(9)	15820(7.4)	
**Calcium or Vitamin D supplement**			0.055			0.248
No	112(91.1)	203608(94.9)		65(97)	200715(93.4)	
Yes	11(8.9)	10893(5.1)		2(3.0)	14159(6.6)	
**Average sleep time (hours) per day**			<0.001			<0.001
≥7	55(44.7)	141352(65.9)		28(41.8)	138860(64.6)	
<7	68(55.3)	73149(34.1)		39(58.2)	76014(35.4)	
**Previous history of fracture**			<0.001			<0.001
No	109(88.6)	208585(97.2)		60(89.6)	211081(98.2)	
Yes	14(11.4)	5916(2.8)		7(10.4)	3793(1.8)	
**Menopause (age, year)**						0.001
<46				2(3.0)	5366(2.5)	
46–50				29(43.3)	57310(26.7)	
>50				7(10.4)	19301(9)	
Pre-menopausal				29(43.3)	132897(61.8)	
**Children to give birth**						0.065
No				7(10.4)	33559(15.6)	
1				17(25.4)	82164(38.2)	
2				33(49.3)	68588(31.9)	
3				9(13.4)	23874(11.1)	
≥4				1(1.5)	6689(3.1)	

Table 5 presented independent risk factors for traumatic foot fractures in adults by gender. For males, alcohol consumption and cigarette smoking increased the risk of foot fracture by 3.00 times (95%CI, 1.90–4.74) and 1.59 times (95%CI, 1.08–2.33), respectively. And compared to those having enough sleep time (≥7h/d), average sleep time <7h/d increased the risk of foot fracture by 2.18 times (95%CI, 1.53–3.13). In addition, history of previous fracture was identified as an independent risk factor for occurrence of foot fractures (OR, 3.82; 95%CI, 2.18–6.69).Similarly for females, alcohol consumption, average sleep time <7h/d and history of previous fracture were identified as independent risk factors for foot fracture and the corresponding OR values were 2.05 (95%CI, 1.14–3.66), 2.25 (95%CI, 1.35–3.75) and 4.78 (95%CI, 2.13–10.73), respectively. Compared with a normal BMI, BMI of 24–27.9 or ≥28 was a risk factor for foot fracture and the corresponding OR values were 2.35 (95%CI, 1.36–4.06) and 3.16(95%CI, 1.46–6.85), respectively. Compared with east region, west region was a protective factor for foot fracture (OR, 0.46; 95%CI, 0.25–0.84).

**Table 5:** Results of multivariate logistic regression of risk factors for foot fractures.

Variables	OR	95%CI		*P*
		Lower limit	Upper limit	
**Males**				
**Alcohol consumption**				
No	Reference			
Yes	2.999	1.898	4.740	<0.001
**Smoking**				
No	Reference			
Yes	1.587	1.080	2.332	0.019
**Sleep time(h/d)**				
≥7	Reference			
<7	2.184	1.527	3.125	<0.001
History of previous fracture				
No	Reference			
Yes	3.815	2.177	6.686	<0.001
**Females**				
**Alcohol consumption**				
No	Reference			
Yes	2.047	1.143	3.664	0.016
**Sleep time(h/d)**				
≥7	Reference			
<7	2.249	1.349	3.748	0.002
**History of previous fracture**				
No	Reference			
Yes	4.779	2.129	10.728	<0.001
**BMI**				
18.5–23.9	Reference			
24–27.9	2.353	1.364	4.057	0.002
> = 28	3.159	1.457	6.848	0.004
**Region**				
East	Reference			
West	0.456	0.246	0.843	0.012

In the final multivariate logistic regression model, the Hosmer–Lemeshow test demonstrated the adequate fitness either for males (X^2^ = 7.483, P = 0.485) or females (X^2^ = 2.733, P = 0.950).

## Discussion

Little information regarding incidence and risk factors for foot fractures is available in the modern literature. This was the first study to show incidence and risk factor of foot fractures from a well-defined population-based survey in China. In the current study, the overall incidence of foot fractures was 39.2/100000/year in 2014, with 50.8/100000/year in males and 27.3/100000/year in females. The most common cause for foot factures was slip, trip or fall, leading to 40.3% of injuries. 63.2% of all foot fractures occurred at home or on the road around. In adults, alcohol consumption, average sleep time <7h/d and history of previous fracture were identified as risk factors for foot fractures in both males and females. Males with cigarette smoking had the 1.59-time increased risk of foot fractures. For females, BMI of more than 24 kg/m^2^ was a risk factor while west region was found to be a protective factor.

In contrast to previous reports, the incidence rate of foot fractures in current study was notably lower. Court-Brown and Caesar [7] reported an incidence of 136.9/100000/year in individuals 12 years or older in Scotland. Curtis et al reported the incidences of individuals aged 18–49 and 50+ years were 121/100000/year and 105/100000 in UK during 1988–2012, respectively. Kannus et al [6] reported that the incidence of fall-induced foot fractures in older people aged 50 years or older increased from 5.6/100000 in 1970 to 15.0/100000 in 2013 and the age-specific incidence was higher in males than females. A UK study showed that incidence of foot of fractures increased from 12.2 during 1990–1994 to 16.1 during 2008–2012 among females, but did not change in males [10]. The present study also showed the male dominance, in accordance with the above studies. The great variation might be explained by geographic or lifestyle differences among regions or countries. Moreover, observational periods, sampling, population size and the exclusion of certain patients might also affect the results. For adult women, slip, trip or fall was the most common mechanism for foot factures, which caused 65.7% of injuries. In contrast, high-energy injures like crushing injury, or fall from heights were more likely to cause the incident foot fracture in adult men. The causal mechanism for foot fractures is in accordance with the place, where individuals work, live and have their daily activities. For males, most of the foot fractures occurred at work place, home and road. For females, the place of foot fracture occurrence was home and road, mostly. In some sense, the type of work such as building could lead to a higher incidence of foot factures for males.

The present study showed that alcohol consumption, average sleep time <7h/d and history of previous fracture increased the risk of foot fractures in both genders. Alcohol consumption has been identified as a recognized risk factor for traumatic fractures in the literature [11, 12]. Scholes et al [12] reported that consuming more than 8 units of alcohol for men or more than 6 units for women on the heaviest drinking day in the past week had a significant independent association with fracture in individuals aged 55 years and over. Excessive alcohol consumption could increase risk for osteoporosis and fracture by the ways of metabolism alcohol-related falls or other trauma. Sleep impairment is well known to be associated with increased injury risk [13, 14]. Stone et al [14] reported that in older women who slept for 5h or less or 5-7h per night were more likely to fall, comparing to those slept for 7-8h per night. Similarly, Holmberg et al [13] reported that sleep disturbance increased fracture risk in most subgroups of fragility fractures in middle-aged men.

A previous history of fracture was a strong risk factor for both males and females and increased 3.82 times and 4.78 times risk of foot fractures in this study. Similar to our finding, a number of previous studies have shown that one fracture often predicts the next [15, 16]. Klotzbuecher et al [15] reported that history of prior fracture at any site is an important risk factor for future fractures in both males and females of all ages. Kanis et al [17] analyzed 15259 men and 44902 women from 11 cohorts and reported that a history of prior fracture is a substantial risk for subsequent fractures. Holmberg et al [13] found that previous fracture was a risk factor strongly associated with low-energy fractures in middle-aged women, but not in men. It can be suggested that patients with a history of previous fracture, especially elderly patients, should be encourage to receive further evaluation for osteoporosis and fracture risk.

In our study, cigarette smoking was identified as an independent risk factor for foot fractures in males, but not in females. Several previous studies have reported that current smoking was associated with a significantly increased risk of fracture [18, 19, 20, 21, 22]. Some studies showed an inverse relation between smoking and BMD. However, how the cigarette smoking influences BMD and fracture risk has not been fully elucidated, although nicotine was recognized to influence bone metabolism directly [19, 22]. The mechanism might also include smoking’s effect on body weight, sex steroid hormone levels, and other hormones and enzymes related to bone regulation. In addition, some researches provided indirect evidence that cigarette smoking might damage the blood supply to bone [21].

Compared to normal BMI, BMI of 24–27.9 kg/m^2^ or more than 28 kg/m^2^ was identified as a risk factor for females in this study. This finding is consistent with results of some previous studies that showed that obese increased risk of fracture in women [12, 23, 24]. Scholes et al [12] found that obesity was associated with higher odds of fracture in women aged 55 years and older. In the meta-analysis of 398610 women conducted by Johansson et al [23], obesity could increase the risk of all osteoporotic fractures and of hip fractures after adjustment for BMD. The accurate mechanism that high BMI increases risk of foot fractures remains unknown, but the excessive loading and increased risk of falls might be involved [25]. Compared with east region, west region was identified as a protective factor for females. Reyes et al [26] reported that in the more affluent areas populations had a higher incidence of hip fracture compared to those living in the deprived areas due to differences in age–sex composition and BMI. We inferred imbalanced development of the economy that the western areas of China lagged behind the eastern areas, and together with the variations in geography, demography and lifestyle, contributed to this result, although the exact reason remains not clear.

The current study is associated with several limitations. First, the incidence rate of foot fractures might be underestimated due to selection effect. Individuals who sustained foot fractures in 2014 might die for some other reasons before the CNFS. Second, as a retrospective survey, this study had its intrinsic weakness in accuracy of data collection, such as lifestyle and dietary information. Combined patients' self-reports and further confirmation by their providing medical records for identification of fracture might partly compensate for the accuracy of case identification.

## Conclusion

Our results indicate the national population-based incidence rate and risk factors for traumatic foot fractures in China, which should be great importance in national healthcare planning and individual health consultation and prevention. It can be suggested that individuals improve their sleep quality and duration, and decrease alcohol consumption to reduce the risk of foot fractures. Individuals should focus more on bone health, active exercises and maintaining normal BMI, especially in those with history of previous fracture, to prevent this injury.

## Supporting information

S1 File Survey Questionnaire. (DOC)
