# Supplementary material for: Incidence and risk factors for foot fractures in China: A retrospective population-based survey
Source: PLoS One. 2018 Dec 26;13(12):e0209740. doi: 10.1371/journal.pone.0209740 (PMC6306245; doi:10.1371/journal.pone.0209740)
Supplement: S1 File — (DOC) [file pone.0209740.s001.doc]

**Number：**

**General information**

**Smoking**  0=Non 1= Average cigarette consumption a day cigarettes，Cumulative years

**Drinking** 0= Non 1= /month， average consumption at a time g or ml， years

**Carbonated drinks** 0= Non 1≥1/D 2≥1/W 3≥1/M 4≤1/M

**Coffee** 0= Non 1≥1/D 2≥1/W 3≥1/M 4≤1/M

**Tea** 0= Non 1≥1/D 2≥1/W 3≥1/M 4≤1/M

**Meat** 0= Non 1≥1/D 2≥1/W 3≥1/M 4≤1/M

**Milk** 0= Non 1≥1/D 2≥1/W 3≥1/M 4≤1/M

**Beans** 0= Non 1≥1/D 2≥1/W 3≥1/M 4≤1/M

Common mode of transportation , How long does it take for a round trip min，Physical exercise h/D，exercise programs

Sleeping h/D，Other diseases ，Course of disease years

Age ，Gender：1=Male 2=Female，Height cm , Weight kg，Blood pressure / , Nationality , Education ， Local residence time years，Whether live alone：0=No 1=Yes

Whether live in a sunny house 0=No 1=Yes，housing type：1.bungalow 2.multistory building 3.high rise building，live in floor, Occupation ，Workplace 1=indoors 2=outdoors，Average working time a day h, working strength ，nature of work：

History of fracture？0= Non 1= Yes；Times of fracture ；Date of fracture： month year

Female：pregnancy delivery ，Menopausal age

Long-used drugs，Drug names：

Calicum/Vitamin D

Family genetic diseases ，Relationship with the patient

Average monthly incomes of the family（Yuan）：0≤999，1=1000-1999，2=2000-4999，3=5000-9999，4≥10000

Suffering from recurrent patellar pain：0= Non 1= Yes，VAS score ；activity with fricative：0= Non 1= Yes；

Morning stiffness of knees：0= Non 1= Yes，Duration min; Metaphysis swelling of knees accompanied with Hyperostosis：0= Non 1= Yes，Diagnosis of Knee osteoarthritis：0= Non 1= Yes（Please fill in the attached table 1）； operation：0= Non 1= Yes， X-ray film：0= Non 1= Yes

Fracture site , date

place , mechanism

Inquirer/Investigator：____________ Survey time：____ __

**编号：**

**全国居民骨折发病率及相关危险因素调查**

**吸烟**  0= Non 1=每天 支， 累计 年;

**饮酒** 0= Non 1= /月， 每次饮酒 g或ml， 度 年

**碳酸饮料** 0= Non 1≥1/D 2≥1/W 3≥1/M 4≤1/M

**咖啡** 0= Non 1≥1/D 2≥1/W 3≥1/M 4≤1/M

**茶** 0= Non 1≥1/D 2≥1/W 3≥1/M 4≤1/M

**肉类** 0= Non 1≥1/D 2≥1/W 3≥1/M 4≤1/M

**牛奶** 0= Non 1≥1/D 2≥1/W 3≥1/M 4≤1/M

**豆类**  0= Non 1≥1/D 2≥1/W 3≥1/M 4≤1/M

常用交通方式 来回需 min，健身 h/D，健身项目

睡眠 h/D，您是否有疾病 ，发病时间 年

年龄 ，性别：1=男2=女，身高 cm 体重 kg，血压 / 民族 

文化程度 ， 本地居住时间 年，独居：0=否 1=是

住房朝阳 0=否 1=是， 居住：1.平房 2.多层 3.高层，住 楼

职业 （**农林牧渔**），工作地点性质 ，您每天在**室内/户外**工作 h

工作强度 等，每天主要从事何种性质的劳动：

既往骨折史？0=否 1=是；骨折 次；具体时间： 年 月

女性：孕 产 ，绝经年龄

有无长期服用药物，药物名称：

Ca/V_D_

家族遗传病 ，与患者关系

家庭人均月收入（元）： 0≤9999， 1=1000-1999， 2=2000-4999， 3=5000-9999， 4≥10000

有无经常反复膝关节疼痛：0=否 1=是，VAS评分 ；活动时有无摩擦音：0=无 1=有；

有无膝关节晨僵：0=无 1=有，时长 min;有无膝关节骨端肥大伴骨质增生：0=无1=有

是否被医生诊断过膝关节骨性关节炎：0=否 1=是（请填附表1）； 是否手术：0=否 1=是

有无X线光片：0=否 1=是

骨折部位 ；骨折发生时间

骨折发生地点 ；骨折原因

调查人：____________ 调查时间：______年______月______日
